# European Immunization Week: two decades of raising awareness and building momentum for the global campaign ahead

**DOI:** 10.2807/1560-7917.ES.2026.31.16.2600335

**Published:** 2026-04-23

**Authors:** Hans Kluge

**Affiliations:** 1 World Health Organization Regional Office for Europe (WHO/Europe), Copenhagen, Denmark

In 1969 I received my first polio vaccination. While mass vaccinations had already brought down numbers of polio cases in my home country Belgium, my parents had seen the immense impact on children affected by the disease and their families, particularly in 1956 when Belgium recorded a peak of over 1,000 cases [1]. Like so many young parents of their time, they wanted to ensure I would be protected.

Just over three decades later, in 2002, the World Health Organization (WHO) European Region was declared polio free [2]. Vaccines had not only wiped out polio, but diphtheria, tetanus, pertussis, measles and rubella incidence had also dropped impressively. Diphtheria cases for example, went down from a high of over 50,000 in 1995 to 897 in 2003. Rubella cases declined from 804,000 in 1999, the first year that numbers were reported to WHO, to 304,000 in 2003 [3]. To achieve these gains, public health efforts had focused on the supply of immunisation, that is, maintaining staffing, cold chains, vaccine roll-out as well as on strengthening surveillance. It was generally assumed that if safe and effective vaccines against dangerous diseases were freely and widely available, people would want them and uptake would be high. However, early warning signs were already emerging, including concerns about the measles-mumps-rubella (MMR) vaccine ignited by an article published in 1998 and retracted later because it falsely linked the vaccine to autism [4]. Many parents who no longer saw these diseases, no longer feared them. The urgency to vaccinate a child on time, or even at all, became less obvious, especially in the following years as misinformation about vaccines was spreading more widely on new and evolving online platforms [5]. Average coverage rates were high, but varied greatly between and within countries in the WHO European Region. However, they had begun to plateau or even decline in some countries; for example, coverage with the first dose of measles-containing vaccine declined in 13 of 53 countries from 2002 to 2003 [3], and outbreaks of measles and rubella showed that pockets of under-vaccinated communities remained, which was illustrated by five countries reporting over 1,000 measles cases and nine countries reporting over 1,000 rubella cases in 2003 [3].

So in 2007, following an initial pilot in 2005, the WHO Regional Office for Europe launched European Immunization Week (EIW) to put more focus on strengthening vaccine uptake, the ‘demand side’ of immunisation. The goal of the initiative, as explained in a 2008 *Eurosurveillance* editorial [6], was not to vaccinate as many people as possible in a single week but, rather, to raise awareness of the importance of immunisation and thereby help sustain vaccine acceptance in the long term. As new vaccines, like the one against human papillomavirus (HPV), were introduced, EIW could also serve as a platform to educate people about them. Target audiences included parents and caregivers, journalists, healthcare professionals and policymakers. A special focus was placed on partnerships, community engagement and activities targeting under-vaccinated groups.

The year 2026 will be the final year of EIW as a standalone campaign, as from 2027, it will be incorporated into World Immunization Week led by the WHO headquarters. It is therefore a good time to reflect on EIW’s legacy and the role of World Immunization Week going forward. Over the 20 years of its existence, EIW became a well-established moment across all 53 countries in the WHO European Region ─ bringing together WHO partners including United Nations Children's Fund (UNICEF), the European Commission, the European Centre for Disease Prevention and Control (ECDC), the European Medicines Agency (EMA) and Rotary, alongside high-level government officials, health authorities, civil society, professional associations and people personally affected by vaccine-preventable diseases. Together, they have been using the week to raise awareness of the importance of immunisation, to educate about specific vaccines and vaccine-preventable diseases and to reduce information and access barriers to vaccination. Each year, the WHO Regional Office for Europe has set out the theme and provided key messages and communication materials, which countries have then translated and adapted for their own audiences.

European Immunization Week has been utilized widely in the Region and in various ways [7], with activities that reach people where they are, rather than only where it is convenient to reach them. In addition to the many press conferences, media workshops, health worker trainings, clinic assessments, countries’ activities have included door-to-door vaccination drives, parliamentary debates, children's theatre, exhibits, community sports events, flash mobs, podcasts, talk shows, university lectures, school visits and vaccination caravans.

Communication campaigns have reached target audiences in every country, through printed materials, radio, television, websites, billboards and social media. Websites launched as part of EIW, such as in Republic of Moldova [8], have continued to provide much-needed information throughout the year.

To address persistent gaps in immunisation coverage, several campaigns and outreach activities have been tailored to reach specific priority groups. For example in 2019, these included journalists in Bulgaria, healthcare workers in Croatia, parents of under-vaccinated children in Cyprus, older adults in Estonia, medical students in Hungary, communities experiencing homelessness and migrants in Kazakhstan, primary school pupils and teachers in Montenegro, Roma communities in Slovakia, nurses in Spain, pharmacists in Turkey and mothers-to-be in Ukraine [9]. What started as a single week of awareness raising in many countries in 2007 has since become something bigger and communicating about immunisation is now recognised as a core part of ongoing immunisation programming in every country in Europe, not a once-a-year occasion.

The ultimate aim of awareness raising was to sustain or improve national rates and help close immunity gaps at community level. Great strides have been made in the intervening years, with vaccinations available against more diseases and progress made since 2007 towards measles, rubella, hepatitis B and cervical cancer elimination. However, while average coverage rates are still high in the Region, some have actually declined since 2007 for example; the third dose of diphtheria, tetanus, and pertussis vaccine declined from 96% in 2007 to 93% in 2024 [3]. Rates also still vary considerably between countries; for example, coverage with the second dose of measles-containing vaccine ranged from 62 to 99% in 2024 [3]. Moreover, rates vary in some countries between sub-national areas. A resurgence of measles in 2024, leading to the highest number of cases ─ over 125,000 [3] in the Region since 1997 ─ demonstrated that an accumulation over time of susceptible individuals in a community, even in countries with high coverage at national level, will eventually lead to outbreaks.

The spread of misinformation has intensified over the past two decades. Fuelled by algorithms that prioritise emotionally charged content and create specialised information environments that reinforce existing scepticism, trust in health authorities and public health measures including immunisation is under strong scrutiny. With advances in artificial intelligence and deepfakes, discerning fact from fiction is becoming ever more difficult for people trying to decide what is best for them and their children.

The COVID-19 pandemic demonstrated that vaccines are a foundational component of national health security rather than a routine item in a health budget. That lesson has sharpened the stakes for decision-makers across Europe. Competing pressures on public health budgets have not gone away, and political commitment to immunisation cannot be taken for granted. European Immunization Week has always provided an opportunity to put that commitment on the political agenda ─ through parliamentary debates, high-level statements, and question and answer sessions with decision-makers ─ and that function matters more now, not less.

The name changes, the work does not. The momentum built, the people engaged, and the awareness raised over two decades jointly with many partners, will endure. Aligning the European campaign with the global one extends the reach of materials and messages while making better use of resources – all in the service of the same goal: trust in health authorities, sustained confidence in vaccines, and the high immunisation uptake that keeps people safer and more secure.

## Note

Hans Kluge is the World Health Organization (WHO) Regional Director for Europe.
